# Raynaud’s Phenomenon with Focus on Systemic Sclerosis

**DOI:** 10.3390/jcm11092490

**Published:** 2022-04-28

**Authors:** Magdalena Maciejewska, Mariusz Sikora, Cezary Maciejewski, Rosanna Alda-Malicka, Joanna Czuwara, Lidia Rudnicka

**Affiliations:** 1 Department of Dermatology, Medical University of Warsaw, Koszykowa 82A, 02-008 Warsaw, Poland; chrabaszcz.magda@gmail.com (M.M.); rosie_alda@msn.com (R.A.-M.); jczuwara@yahoo.com (J.C.); lidiarudnicka@gmail.com (L.R.); 2 National Institute of Geriatrics, Rheumatology and Rehabilitation, Spartańska 1, 02-637 Warsaw, Poland; 3 1st Department of Cardiology, Medical University of Warsaw, 02-091 Warsaw, Poland; cmaciejewski6@gmail.com

**Keywords:** Raynaud’s phenomenon, microcirculation, systemic sclerosis, vasculopathy, capillaroscopy, iloprost, alprostadyl, sulodexide

## Abstract

Raynaud’s phenomenon is a painful vascular condition in which abnormal vasoconstriction of the digital arteries causes blanching of the skin. The treatment approach can vary depending on the underlying cause of disease. Raynaud’s phenomenon can present as a primary symptom, in which there is no evidence of underlying disease, or secondary to a range of medical conditions or therapies. Systemic sclerosis is one of the most frequent causes of secondary Raynaud’s phenomenon; its appearance may occur long before other signs and symptoms. Timely, accurate identification of secondary Raynaud’s phenomenon may accelerate a final diagnosis and positively alter prognosis. Capillaroscopy is fundamental in the diagnosis and differentiation of primary and secondary Raynaud’s phenomenon. It is helpful in the very early stages of systemic sclerosis, along with its role in disease monitoring. An extensive range of pharmacotherapies with various routes of administration are available for Raynaud’s phenomenon but a standardized therapeutic plan is still lacking. This review provides insight into recent advances in the understanding of Raynaud’s phenomenon pathophysiology, diagnostic methods, and treatment approaches.

## 1. Introduction

Raynaud’s phenomenon (RP) is defined as intermittent, excessive vasoconstriction of the microvasculature, triggered by cold exposure or emotional stress [1]. The classic clinical picture involves changes in skin colour from white (ischemia), to blue (cyanosis), and red (reperfusion). These changes are associated with a significant burden of pain and hand-related disability [2]. Raynaud’s phenomenon most often occurs in the fingers/toes. Less commonly, the nose, tongue, nipples, and pinnae of the ears may be involved [3].

Raynaud’s phenomenon can be subdivided into primary (idiopathic) and secondary forms [Table 1]. Primary Raynaud’s phenomenon (PRP) has an estimated prevalence of 5% in the general population, and most often occurs in young women [3]. Both forms of Raynaud’s phenomenon are more common in cold climates [4].

Patients with PRP have a younger age of onset (usually between the age of 15 and 30) than those with secondary Raynaud’s phenomenon (SRP), and the thumb is usually not involved [5]. The latest diagnostic criteria for PRP are: history of episodic, acral, bi- or triphasic colour change; normal nailfold capillaries; antinuclear antibodies (ANAs) titer < 1:40 (i.e., negative); no association with underlying systemic disease; and no history of collagen vascular disease [6]. 

A large population-based cohort study revealed that low body weight and previous involuntary weight loss are significantly associated with an increased risk of RP in both men and women [7].

Symptoms of SRP depend on the frequency, severity, and duration of blood vessel spasm. Episodes usually last minutes but may last several hours, potentially causing digital ulceration, irreversible ischemia, necrosis, and secondary infection. Consequently, timely and accurate recognition of SRP is crucial as it alters patient management and prognosis [4]. 

**Table 1:** Differences between primary and secondary Raynaud’s phenomenon [3,5,6,8,9]; ANA—antinuclear antibodies.

Raynaud’s Phenomenon	Primary	Secondary
Age at Onset	usually between 15 and 30 years	over the age of 40
ANA	negative or low titre	often positive
Change of Microcirculation	functional vascular abnormalities	functional and structural microvascular changes
Pain or Paresthesia	rare	often
Capillaroscopy	normal capillaroscopic pattern	abnormal capillaroscopic pattern
Course	complete reversibility of episodic digital ischemia	can result in digital ulceration, irreversible ischemia and necrosis
Peripheral Pulses	strong and symmetrical	dependent
Swollen (“puffy”) fingers	no	yes
Digital Ulcers	no	common

**Figure 1:**
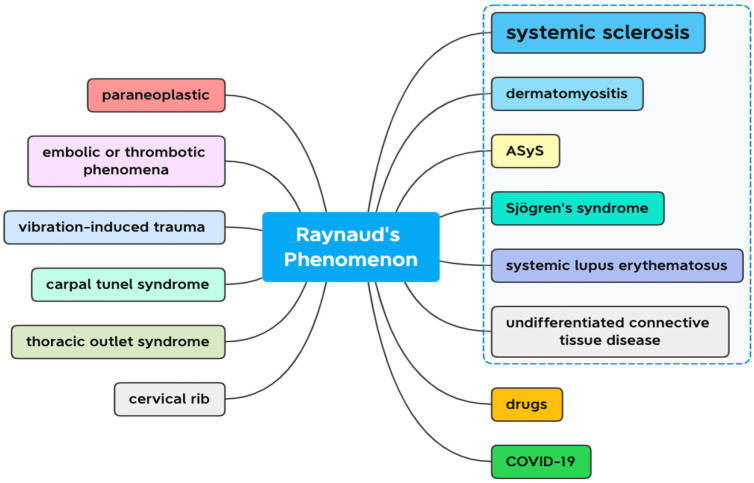
Secondary causes of Raynaud’s phenomenon. Blue dashed line—connective tissue diseases; ASyS—antisynthetase syndrome.

Secondary causes of RP (Figure 1) include various autoimmune connective tissue disorders—systemic sclerosis (SSc), systemic lupus erythematosus (SLE), Sjögren’s syndrome, idiopathic inflammatory myopathies, antisynthetase syndrome (ASyS), thoracic outlet syndrome; cervical rib, embolic or thrombotic events; vibration-induced trauma; and multiple different medications [2,10,11]—the most relevant being β-adrenoceptor blockers, vinyl chloride, interferons, and chemotherapy [12,13]. Raynaud’s phenomenon frequently represents the initial manifestation in patients who have mixed connective tissue disease (MCTD). It is the cutaneous symptom of a systemic vasculopathy that is characterized by intimal fibrosis and blood vessel obliteration that frequently leads to visceral involvement. Raynaud’s phenomenon appears in 18–46% of patients with systemic lupus erythematosus [14,15]. Looking for signs of arthritis or vasculitis, as well as a number of laboratory tests (Table 2), may separate them. Complete blood count may reveal a normocytic anaemia, suggesting chronic disease or kidney failure. Blood tests for urea and electrolytes may reveal kidney impairment. Tests for rheumatoid factor, erythrocyte sedimentation rate, C-reactive protein, and autoantibody screening may reveal specific causative illnesses or an inflammatory process. Thyroid function tests may reveal hypothyroidism [16,17].

Systemic sclerosis is an autoimmune disorder characterized by inflammation, fibrosis, and microvasculopathy. It results in potentially widespread fibrosis and vascular abnormalities, which can affect the skin, lungs, heart, gastrointestinal tract and kidneys. The uncontrolled fibrosis of the skin and internal organs in systemic sclerosis leads to severe and sometimes life-threatening complications.

The underlying mechanisms are complex and remain largely unknown [18,19]. Definitive diagnosis is made with fulfilment of the 2013 European League Against Rheumatism (EULAR) and American College of Rheumatology (ACR) classification criteria [20]. Over the past 15 years, efforts have been made towards early diagnosis [21]. Almost all individuals with SSc have detectable circulating antibodies against nuclear proteins and different SSc phenotypes are strongly associated with the different antibody types [22]. Several forms of the disease have been esteemed. Diffuse cutaneous systemic sclerosis (dcSSc) is characterized by the quickest course, with internal organ involvement already at early disease stage, and poor prognosis [23]. Skin thickening confined to sites above the elbows or knees is classified as limited cutaneous SSc (lsSSc). Around 20% of patients with lcSSc may present with features of SSc as a component of the overlap syndrome [24,25]. 

Almost all SSc patients suffer from RP, typically the initial manifestation of disease and may precede the involvement of other organs by many years, especially in lcSSc [26]. There are many areas in which RP initiates considerable disease-related morbidity in SSc patients, including impaired hand function, pain, reduced social engagement, diminished body image, increased dependence on others, and reduced quality of life [27]. Though rare, several paraneoplastic causes of RP have been identified secondary to malignancies of the lung, breast, uterus, and ovaries [28,29]. 

RP has additionally been reported as a side effect of biological agents—for example interferon, radiotherapy, and chemotherapeutic agents—particularly bleomycin (alone or combined with vinca alkaloids or cisplatin) and cisplatin (combined with other chemotherapy agents); beta-adrenergic blocking agents may also provoke paroxysmal vasospasm of small vessels [6].

Kim et al. reported a case of RP in a 70-year-old woman, with no history of connective tissue disease, secondary to pembrolizumab therapy for gallbladder cancer [30]. A further case report on the development of IL-17A antagonist (secukinumab)-related RP in a 35-year-old female patient with ankylosing spondylitis has also been described [31]. Additionally, Bouaziz et al. described a patient with proven COVID-19 infection presenting with RP and chilblain appearance of the hands [32].

## 2. Pathophysiology

The pathophysiological mechanisms behind RP are not entirely understood; generally, it is characterized by excessive vasoconstriction of the digital arteries, precapillary arterioles, and cutaneous arteriovenous anastomoses [33]. 

In PRP, vasospasm of the digital and cutaneous vessels is believed to occur as a consequence of an increased alpha_2c_-adrenergic response, and does not result in vascular pathology [34]. Ascherman et al. put forward an autoimmune etiology, proposing cytokeratin 10 (K10) as a potential autoantigen. Their study on mice showed that anti-K10 antibodies can mediate ischemia, similar to that seen in primary Raynaud’s Phenomenon [35].

In SRP, affected endothelial cells exhibit amplified exocytosis of endothelin-1 and ultra-large von Willebrand factor (ULVWF), which contribute, respectively, to increased vasospasm and capillary thrombosis. In addition, it is believed that increased transforming growth factor β (TGFβ), endothelin-1, cytokines, and angiotensin II drive the process of myofibroblast proliferation, vascular fibrosis and dropout in SSc patients [34]. Nitric oxide (NO) has a complex role in the disease process [36]. A decrease in endothelial formation of NO results in diminished vascular relaxation and extended vasoconstriction. Conversely, overproduction of NO leads to increased generation of reactive oxygen species and plays a pathogenic role in fibrosis. 

Gualtierotti et al. found that markers of endothelial damage are regularly elevated in patients with PRP at their first assessment, even when there are no capillaroscopic abnormalities or autoantibodies detectable. They are particularly increased in patients with very early SSc. The plasma concentration of tissue-type plasminogen activator (t-PA) and von Willebrand factor (vWF)—two markers of endothelial damage, as well as interleukin-6 (IL-6)—a pro-inflammatory cytokine, were evaluated. After a 36-month follow-up, those with higher basal concentrations of markers of endothelial damage had developed connective tissue disease. Von Willebrand factor analysis showed clear differences between primary and secondary RP patients. These findings suggest that markers of endothelial damage are elevated in RP patients who go on to develop SSc or other connective tissue diseases, even in the absence of capillaroscopic abnormalities [37].

A recent study by Taher et al. demonstrated use of a non-invasive NO-dependent method to identify peripheral microvascular endothelial dysfunction in patients with SRP. The association between SRP and microvascular peripheral endothelial dysfunction was also significant after adjusting for confounding variables, including conventional risk factors for cardiovascular disease, and vasoactive medications. This also remained significant in women after stratifying only by sex. It was emphasized that detection of microvascular peripheral endothelial dysfunction at an early stage could help to identify individuals with SRP who are at risk of developing connective tissue disease, as well as cardiovascular disease. Early detection could additionally indicate who may benefit from frequent screening, prompt initiation of preventative treatments, and modification of risk factors [38].

## 3. Genetics

A genetic predisposition for RP has been demonstrated in two studies demonstrating greater concordance amongst monozygotic than dizygotic twins. Heritability for RP is reported to be 55–64% [39,40]. 

Polymorphisms in various genes encoding ion channels or vasoactive agents have been hypothesized to result in the RP phenotype. It is suggested that genetic variation in temperature-responsive or vasospastic genes may underlie RP manifestation. Several studies have investigated candidate genes that could potentially regulate vascular reactivity [41,42]. Munir et al. aimed to evaluate the association between RP and single nucleotide polymorphisms (SNPs). Temperature-sensing receptor channels called thermo-sensitive transient receptor potential (TRP) ion channels include TRPA1 and TRPM8. These are cold-sensing and have been proposed to mediate cold-induced vascular responses in skin in vivo. This is linked, at least in part, to the expression of these channels on perivascular sensory nerves [41]. Calcitonin-related polypeptides, alpha and beta (CALCA, CALCB), encode the peptide hormones calcitonin, calcitonin gene-related peptide, and katacalcin by tissue-specific alternative RNA splicing of gene transcripts and cleavage of inactive precursor proteins. Calcitonin is involved in the regulation of calcium levels and phosphorus metabolism. Calcitonin gene-related peptide functions as a vasodilator [43]. NO derived from neuronal nitric oxide synthase (nNOS) facilitates the restorative vasodilator response after cold exposure; thus, the gene encoding nNOS (NOS1) has also been investigated [41]. Munir et al. found that one polymorphic variant within the NOS1 gene was significantly associated with RP in the general population [42]. 

## 4. Diagnosis

A detailed medical history, laboratory tests, and nailfold capillaroscopy form the basis of RP diagnosis [17]. Follow-up nailfold capillaroscopy should be performed every 12 months in patients with significant nailfold videocapillaroscopy disturbances present at baseline [44]. Laboratory investigations should comprise a full blood count, inflammatory markers, thyroid function, and ANA testing by indirect immunofluorescence (accompanied by ELISA or solid-phase immunoassays to determine antigen specificities where possible). A negative ANA with cytoplasmic stain could indicate anti-synthetase antibodies, such as anti-Jo-1, or rarer SSc-specific autoantibodies such as anti-eukaryotic initiation factor 2B autoantibodies (anti-EIF2B) [45].

## 5. Capillaroscopy

Nailfold capillaroscopy is a simple, non-invasive technique that allows both qualitative and quantitative evaluation of the microcirculation, thus enabling early detection of abnormalities. At the nailfold, capillaries are positioned parallel to the surface of the skin, allowing full morphological assessment [46,47]. Among the most important indications for capillaroscopy are the differential diagnosis of primary and secondary RP. 

**Figure 2:**
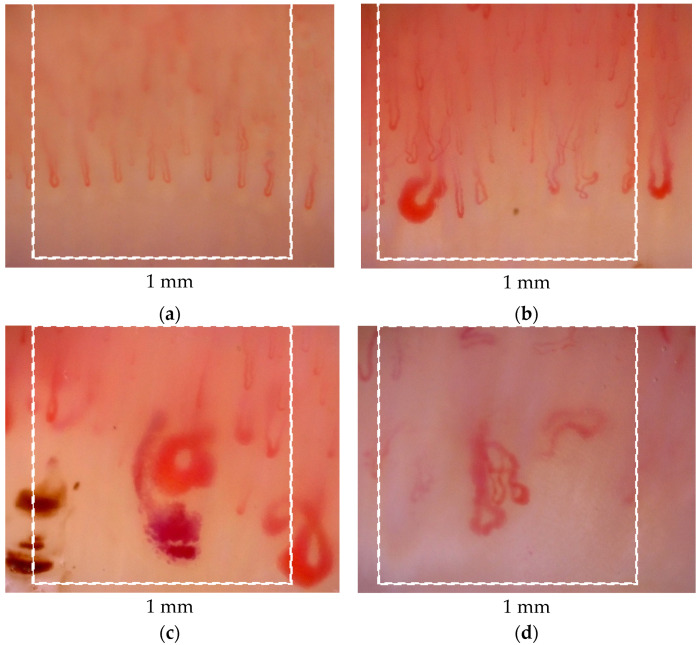
Nailfold videocapillaroscopic (×200) patterns of microangiopathy: (**a**)—normal; capillaroscopic characteristics: density—normal, 8 capillaries in 1 linear mm; dimension: within normal limits; morphology: normal shapes of capillaries; haemorrhages: absent (**b**)—early; capillaroscopic characteristics: density—7 capillaries in 1 linear mm; dimension: presence of giants (homogeneous enlargement of all three limbs of the capillary with the diameter ≥50 µm); morphology: hairpin shaped capillaries; haemorrhages: absent (**c**)—active; capillaroscopic characteristics: density—lowered, 4 capillaries in 1 linear mm; dimension: presence of giants (homogeneous enlargement of all three limbs of the capillary with the diameter ≥50 µm); morphology: presence of abnormally shaped capillary; haemorrhages: present (**d**)—late capillaroscopic characteristics: density—lowered, 2 capillaries in 1 linear mm; dimension: not measured because of presence of abnormal shape; morphology: presence of abnormally shaped capillary; haemorrhages: absent.

Capillaroscopy is included in the 2013 American College of Rheumatology (ACR)/European League Against Rheumatism (EULAR) recommendations [20]. It is considered a key investigation in both the very early phases of the disease, and in monitoring disease progression (Figure 2). Cutolo et al. proposed three progressive capillaroscopic patterns in SSc—‘early’, ‘active’, and ‘late’. The ‘early’ pattern is defined as the presence of a few giant capillaries, single microhemorrhages, and preservation of capillary architecture without capillary loss. ‘Active’ presents as numerous giant capillaries and microhemorrhages, mild disturbance of the capillary architecture and moderate capillary loss. The ‘late’ pattern is characterized by severe capillary loss with extensive avascular areas, disorganization of the capillary architecture and ramified/bushy capillaries [48]. 

A clinical expert-based, fast track decision algorithm was developed to facilitate differentiation of a “non-scleroderma pattern” from a “scleroderma pattern” on capillaroscopic images. The algorithm demonstrated excellent reliability when used by capillaroscopists with varied expertise levels compared to principal experts, and corroborated with external validation [49,50].

In addition capillaroscopic changes have been observed in dermatomyositis, polymyositis, antiphospholipid syndrome, Sjogren’s syndrome, and systemic lupus erythematosus [51]. Dermatomyositis pattern, often associated with aspects of the SSc pattern, includes the presence of two or more of the following findings in at least two nail folds: enlargement of capillary loops, loss of capillaries, disorganization of the normal distribution of capillaries, ‘budding’ (‘bushy’) capillaries, twisted enlarged capillaries, and capillary haemorrhages (extravasates) [51,52]. Characteristic systemic lupus erythematosus pattern includes morphological alterations of capillary loops, venular visibility and sludging of blood with variability in capillary loop length [53]. Capillaroscopic abnormalities in SS ranged from non-specific findings (crossed capillaries) to more specific findings (confluent haemorrhages and pericapillary haemorrhages) or SSc-type findings [54,55]. Multiple hemorrhages from normal-shaped capillaries, which appear parallel/linear and arranged perpendicularly to the nailfold bed, are called “comb-like” hemorrhages and are suggestive of antiphospholipid syndrome [56]. 

It has been shown that the ability to detect capillary abnormalities increases as the number of fingers examined increases. Sensitivities ranged from 31.7% to 46.6% for only one finger (right middle and left ring finger, respectively), 59.8% for both ring fingers, 66.7% for a four-finger combination (both ring and middle fingers) and 74.6% for the eight-finger standard. In order to achieve the most accurate assessment during routine capillaroscopic examination, all eight nailbeds should be examined omitting the thumbs, where it is more difficult to visualize and classify capillaries [57,58]. It should be noted that in a time pressured scenario, the best two-finger combination to detect capillary abnormalities is both ring fingers [58]. 

Nailfold videocapillaroscopy is the standard, although a handheld dermatoscope or an ophthalmoscope may also be used as screening tools [50]. The nailfold videocapillaroscopy technique with 200× magnification, capturing at least two adjacent fields of 1 mm in the middle of the nailfold finger, is the standard capillaroscopic technique to perform nailfold capillaroscopy [50].

Ideally all dermatology specialists should have access to videocapillaroscopy; a pragmatic solution for practitioners may be to have a low-cost capillaroscopy system. Technologies using a smartphone camera could help to improve availability to nailfold capillaroscopy whilst still providing accurate results [59]. Research regarding automated measurement of capillaroscopic characteristics is currently under way and holds promise as an objective clinical outcome measure [50]. Interestingly, a consensus-based assessment of dermatoscopy versus nailfold videocapillaroscopy by a European League against Rheumatism study group revealed tenuous promise for dermatoscopy as a tool for the initial screening of nailfold capillaries in RP. However, as perhaps expected, dermatoscopy is less sensitive, but more specific, in regard to detecting abnormalities, compared with videocapillaroscopy [60]. 

Qualitative analysis is subjective, and quantitative analysis is time-consuming when done manually. A study performed by Cutolo et al. accomplished validation of fully automated AUTOCAPI software for measuring the absolute capillary number over 1 linear/mm in NVC images. The software was subsequently optimized to assess capillary number in the shortest possible time and with the lowest possible error, in both healthy subjects, and those with SSc [61]. 

## 6. Laser Doppler Flowmetry 

Laser Doppler flowmetry (LDF) is a semi-quantitative imaging technique useful for studying the nitric oxide endothelial-dependent vascular response and axon reflex-mediated vasodilation. Impaired regulation of NO vascular tone has been described in patients with SSc-associated RP when compared to those with PRP and healthy controls [62,63]. Laser Doppler flowmetry has been proposed as a method for evaluating blood perfusion of the skin. This is a functional assessment of the vessels of the skin, involving the deeper dermal vessels in addition to the capillaries [64]. 

Melsen et al. completed a systematic review evaluating the use of LDF, describing the results of quality reports on assessment of the skin’s microcirculatory flow at the level of the fingertip in SSc patients, and investigating the validation status of LDF as an outcome measure. The systematic review highlights the very preliminary validation status of LDF in the assessment of the microcirculatory flow in SSc [65].

In a study performed by Gregorczyk-Maga et al., LDF was used to investigate oral capillary flow in PRP patients who habitually have dysfunction in the microcirculation of the oral mucosa and who often have lesions in the oral cavity [66].

Time to postocclusive peak blood flow measured by LDF is an extremely accurate test for distinguishing patients with PRP from healthy controls [67].

An additional study performed by Waszczykowska et al. presented the suitability of LDF for assessment of the degree of microangiopathy present in SSc patients. Assessment of the skin perfusion value in SSc patients should on the basis of parameters obtained during microcirculation challenge tests [68].

## 7. Thermography

Thermal imaging is an indirect method that makes use of a thermal camera to image skin temperature and demonstrate underlying blood flow [69]. Thermal imaging has been used to evaluate RP in several studies; the response to lower temperatures was able to differentiate between PRP and RP secondary to SSc [70]. 

Patients with SSc-related RP have been found to have structural changes in the digital arteries and microcirculation with a decrease in baseline blood flow. This typically does not return to normal after a cold challenge with rewarming, in direct contrast to primary RP, in which the fingers classically rewarm [71]. 

Measurements made by mobile phone thermography compared favorably with those made by standard thermography, paving the way for ambulatory monitoring in non-controlled environments; this will enable further assessments to increase the understanding of RP episodes [69]. Infrared thermography may additionally be a method of verification in Raynaud’s Phenomenon [72]. 

## 8. Laser Speckle Contrast Analysis (LASCA)

Laser speckle contrast analysis (LASCA) is a tool used to investigate variations in peripheral blood perfusion during long-term follow-up and can safely monitor the evolution of digital ulcers in SSc patients [73].

LASCA can quantify blood flow over a defined area and is based on the concept that when laser light illuminates a tissue it forms a speckle pattern. Variations in this pattern are analyzed by dedicated software—static areas demonstrate a stationary speckle pattern, in contrast with mobile objects—such as red blood cells—that cause the speckle pattern to fluctuate and appear blurred. The amount of blurring (contrast) is analyzed and thus interpreted as blood perfusion [74].

The pilot study completed by Ruaro et al. determined that the hand blood perfusion, as evaluated by LASCA, was lower in PRP than in SSc patients with the “early” nailfold videocapillaroscopy microangiopathy pattern [75].

## 9. Treatment

Lifestyle modifications are essential in all patients with RP [8]. Patients’ education is an important aspect of disease management and patients’ support organizations provide them with valuable education on the topic [76]. The first line of the treatment is based on avoiding triggering factors such as: exposure to cold, sudden changes of temperature, stress, cigarette smoke, and infections [34]. Patients should dress warmly (including warm gloves and socks). A number of different types of gloves have been proposed for patients with RP to reduce the risk of attacks, including battery-heated and specifically ceramic-impregnated gloves [77]. During the vasospasm, it is advised that one should place one’s hands under warm running water or to rub one hand against the other to intensify blood flow [78]. Because stress may trigger an attack, learning to recognize and avoid stressful situations may help control the number of attacks. Exercise can improve circulation, among other health benefits. Avoidance of repeated trauma to the fingertips by all patients with RP and avoidance of vibrating tools utilization by patients with vibration-induced RP has to be underlined [79]. Patients should be counselled regarding the critical importance of smoking cessation as nicotine enhances vasoconstriction [80]. Certain medications such as beta-blockers, ergotamine, or sumatriptan, and some types of chemotherapy, specifically, cisplatin and bleomycin, were most likely to induce the Raynaud’s phenomenon. If possible, alternative therapies that do not alter peripheral blood flow should be considered [12]. In most cases of primary Raynaud’s phenomenon, lifestyle modifications may be sufficient to control the symptoms [17,81].

Pharmacological treatment is required when adaptive measures to avoid cold exposure are ineffective. RP reflects excessive vasoconstriction; thus, vasodilator therapy—particularly targeted to the cutaneous circulation—is a major focus. Patients with connective tissue disease-associated RP, SSc in particular, may progress to tissue injury; hence, drug treatment often needs to be more ‘aggressive’ to prevent/minimize tissue loss [Table 3].

## 10. Calcium Channel Blockers

Calcium channel blockers (CCBs) are generally considered to be the first-line pharmacotherapeutic treatment of PRP, and are the group of drugs which have been most extensively researched. According to the 2017 update of the European League against Rheumatism (EULAR) recommendations for the treatment of SSc, oral therapies with CCBs are strongly recommended (strength of recommendation A) [16,82]. CCBs are currently the most frequently prescribed drug for PRP. Nifedipine and amlodipine are considered to be the most effective agents, blocking calcium channels located in the cell membranes of vascular smooth muscle and cardiac muscle. Consequently, calcium ion entry into cells is inhibited, resulting in blood vessel relaxation and improved blood supply to tissues [83]. Doses should be adjusted depending on individual tolerance, with particular caution advised in patients with low arterial blood pressure [84].

A meta-analysis of randomized clinical trials concluded that CCBs are only somewhat effective at reducing the frequency of Raynaud’s attacks in PRP [42,85]. Whereas other studies suggest that CCBs may be effective at decreasing the severity of attacks, pain and disability associated with RP [83].

## 11. Phosphodiesterase-5 (PDE-5) Inhibitors

Phosphodiesterase-5 (PDE-5) inhibitors are commonly used as a second-line systemic agent to manage RP resistant to CCBs. Inhibition of PDE-5 activity allows accumulation of cGMP within endothelial cells, which alters the cellular response to prostacyclin or nitric oxide, and in turn dilates blood vessels [86].

A 2013 meta-analysis of six randomized controlled trials including 296 SRP patients revealed a significant, moderate effect on the clinical severity, duration, and frequency of attacks [87]. Additionally, a significant decrease in the number of digital ulcers in SSc patients with RP was found in a randomized, placebo-controlled study in patients receiving sildenafil compared to a placebo [88].

Adverse effects of these PDE-5 inhibitors include flushing, headaches, and dizziness. Less common side effects include hypotension, arrhythmias, cerebral vascular accidents, and vision changes [89]. 

## 12. Prostaglandin Analogs

While oral prostaglandins have not shown any benefit in RP, prostacyclin analogs administered intravenously exhibit a strong vasodilative effect which considerably improves the clinical condition, particularly among patients with ulcers and erosions [90]. 

Iloprost is a synthetic analogue of prostacyclin (PGI2), with vasodilatory and antiplatelet effects; however, it is more stable than PGI2, has a longer half-life (20 to 30 min) and better solubility [91]. Iloprost activates PGI2 receptors, thus stimulating adenylate cyclase to generate cyclic adenosine monophosphate (cAMP). PGI2 receptors inhibit vascular smooth muscle constriction and platelet aggregation. They are also expressed on endothelial cells, where they initiate multiple protective effects, including amplification of endothelial adherens junctions and decreased monolayer permeability [92]. Iloprost infusions are frequently recommended as second-line treatment after CCBs and are the first-line therapeutic choice for digital ulcerations and critical ischemia [21,93]. A meta-analysis determined that the use of iloprost in critical limb ischemia was effective in improving ulcer healing, relieving pain, and reducing the need for amputations [94]. In cases of pre-existing digital ulcerations, iloprost promotes healing and reduces the incidence of new ulcerations [16]. Three further studies described an improvement in nailfold microvascularization following iloprost treatment [95]. 

In 2017, the EUSTAR recommendations allocated intravenous iloprost a Grade A recommendation for management of severe SSc-related RP attacks and for digital ulcer treatment [16]. However, in the recommendations, the dosing and therapeutic regimen was not specified. The absence of an accepted regimen is a major impediment to the administration of iloprost in SSc. According to the Delphi concensus, intravenous iloprost can be useful in RP that is severe or refractory to CCB and PDE-5i. To control symptoms, it is recommended that iloprost be administered 1–3 days every month. Dosing should be determined according to the tolerance, starting from 0.5 up to 3.0 ng/kg/min. To achieve a lasting effect, infusions must be repeated regularly [16,96,97]. Interestingly, the pharmacological actions may persist longer than suggested by the pharmacokinetic profile (i.e., weeks to months) [98]. 

Currently, intravenous iloprost is available in several countries only for RP secondary to SSc for a duration of 3–5 days. For RP and digital ulcer healing, expert consensus proposes a regimen of 1–3 days per month, with 1 day per month for DU prevention. These recommendations allow clinicians some scope on how to personalize intravenous iloprost therapy according to patients’ needs [93]. However, although these suggestions are supported by an expert group for use in a clinical setting, it would be necessary to formally validate the recommendations in future clinical trials.

As iloprost is not available in some countries, alprostadil (a combination of prostaglandin E1 with a-cyclodextrin in a 1:1) has been found to be an effective alternative for SRP [99]. Alprostadil is primarily used to maintain patency of the ductus arteriosus, and also has mild pulmonary vasodilatory effects. It reportedly inhibits macrophage activation, neutrophil chemotaxis, and release of oxygen radicals and lysosomal enzymes. It influences coagulation by inhibiting platelet aggregation and potentially by inhibiting factor X activation. Alprostadil may promote fibrinolysis by stimulating production of tissue plasminogen activator. The overall benefits of iloprost and alprostadil are comparable, without significant differences in clinical efficacy or circulating markers of endothelial damage [100].

Epoprostenol, the first prostacyclin agent approved by the US Food and Drug Administration (FDA), in 1995, requires continuous intravenous infusion via a dedicated central venous catheter with infusion pump. Epoprostenol stimulates vasodilation of pulmonary and systemic arterial vascular beds and impedes platelet aggregation [11,101]. Based on published evidence, the initial dose of intravenous epoprostenol for treatment of refractory RP, with or without ischemic ulcers, should not exceed 2 ng/kg/min. A conservative titration schedule, based on those used in previous studies, should allow for rate increases of 1 ng/kg/min every 15 min as tolerated, adjusted as per the onset of treatment-emergent adverse effects. It should be noted that more aggressive uptitrations of 2 to 2.5 ng/kg/min were used in some studies. However, as a consequence of the lack of standardized efficacy outcomes in the available literature, it is not possible to assess if such regimens hold any advantages other than reaching the maximum dose more quickly. Intermittent infusions of 5 to 6 hours’ duration should be initially considered to limit drug exposure and potential toxicities. However, it is reportedly reasonable to use continuous infusions for up to 72 h in patients unresponsive to intermittent therapy [101].

Epoprostenol is contraindicated in patients with congestive heart failure due to left ventricular dysfunction, and in those with known history of hypersensitivity reactions to the drug. Other adverse effects that should be monitored for include pulmonary edema, hemodynamic instability, line infections, and bleeding [101].

## 13. Endothelin Receptor Antagonists

Bosentan is an endothelin receptor antagonist (ERA) primarily used to manage severe pulmonary hypertension. A starting dose of 62.5 mg twice a day for four weeks, followed by 125 mg twice a day for 12 or 20 weeks, has shown some effectiveness in preventing formation of new digital ulcers, but did not influence healing of pre-existing ulcers [102,103]. Potential adverse effects include headaches, dizziness, and hypotension [16]. Adverse drug reactions are relatively mild, but during the treatment monthly liver function and 3-monthly full blood count is required [104,105].

## 14. Angiotensin II Receptor Blockers

Angiotensin II receptor blockers (ARBs) are reserved as a third-line treatment for mild Raynaud’s phenomenon. There has been only one trial including 52 patients (25 patients with primary RP and 27 with SSc-related RP); this was an open-label, unblinded, controlled trial, during which a 12-week treatment with losartan 50 mg/day resulted in reduced frequency and severity of RP attacks in comparison to nifedipine 40 mg/day. The benefit was more noticeable in the subgroup of patients with primary RP. Losartan is widely available, accessible, and has an acceptable side effect profile [82,106,107].

## 15. Sulodexide

Sulodexide is a safe rheological drug used successfully as a supportive way of treating RP [84]. It consists of a purified mixture of glycosaminoglycans acquired from bovine intestinal mucosa, comprising a heparin of a rapidly moving field of electrophoresis (80%) and dermatan sulfate (20%). It functions as an anticoagulant, is pro-fibrinolytic and anti-inflammatory, disrupts the process of fibrosis, and has a protective influence on vascular endothelial cells. Due to its pleiotropic activity and high safety profile, the benefits from sulodexide may be applied to many dermatological diseases [108]. 

SSc patients with secondary microcirculatory disorders who are intolerant to prostanoids, where there are contraindications, may be treated with sulodexide, 600 lipasemic units (LSU) intravenously twice a day. This dosing regimen has previously produced good therapeutic results. Other than sporadically observed dizziness and hypotension, no significant side effects were noted. In patients’ pre-existing digital erosions and ulcers, a 3–4 day cycle of intravenous sulodexide, at 600 LSU twice a day every 4–6 weeks, has been shown to improve lesion healing [109]. 

Results of a recent pilot study suggest that the use of sulodexide treatment in RP results in a long-term improvement of capillary flow, a decrease in episode recurrence, and a reduction in pain intensity [110].

During parenteral treatment with sulodexide, it is imperative to discontinue any use of heparin or oral anticoagulants to reduce the bleeding risk [109]. 

## 16. Statins

Statins, 3-hydroxy-3-methylglutaryl coenzyme A (HMG-CoA) reductase inhibitors, are extensively used to reduce serum cholesterol levels in the primary and secondary prevention of cardiovascular disease. Statins also have direct, vasculoprotective effects, which are independent of their ability to lower circulating LDL levels. Statins may be beneficial in RP patients, including those with SSc. Statins increase endothelial nitric oxide synthase expression and thus nitric oxide production, decrease oxidative stress, reduce endothelin-1 expression, impede endothelial apoptosis, increase endothelial progenitor cell mobilization, and promote microvascular growth [111,112]. Statins additionally inhibit endothelial–mesenchymal transition, which may contribute to vasculopathy and tissue fibrosis in SSc [113]. In a study of SSc patients, treatment with a statin was found to reduce the severity of RP vasospastic episodes and improved endothelial function. This was associated with increased levels of nitric oxide, reduced oxidative and inflammatory stress, increased quantity of endothelial progenitor cells, and amelioration of circulating concentrations of von Willebrand factor [114]. Data from a study performed by Abou-Raya et al. suggest that atorvastatin may exert beneficial effects in SSc by protecting the endothelium and improving its functional activity [115]. 

## 17. Topical Vasodilators

Topical vasodilators may be used as adjuvant therapy for RP patients. 10% nifedipine cream and 10% nitroglycerin gel have both been accepted as efficient therapeutic options with side-effects comparable to placebo usage. Local topical nitrates display significant efficacy in treatment of both primary and secondary RP [116]. Topical nitrates have been reported to increase perfusion at both distal digital ulcer and extensor digital ulcer cores in SSc patients, when compared to a placebo and evaluating with laser doppler imaging [117]. Wortsman et al. found that 5% sildenafil cream significantly improved blood flow in digital arteries (an increase of 9.2 mm/s, *p* < 0.0083). A trend toward improvement was also observed for vessel diameter in patients with SRP (*p* = 0.0695), suggesting local vasodilatation. Adverse effects to topical vasodilators include headaches and dizziness—no serious adverse effects were detected [118]. A study by Bentea et al. assessed the effects of nitroglycerin patch application to the dorsum of the hand. Results showed an increase in blood flow and hand temperature in patients with SSc after a cold challenge using laser doppler imaging [119]. 

## 18. Sympathectomy

Surgical treatment options involving sympathectomy or arterial reconstruction may be required in patients who suffer from incapacitating pain and ulcers with torpid evolution. However, these techniques carry the risk of comorbidities and may not always provide satisfactory results.

Cautious selection of RP patients is necessary, and endoscopic thoracic sympathectomy should be reserved as an ultimate choice only for patients who have severe symptoms that are treatment-resistant with serious complications and impaired quality of life. The limiting factor with sympathectomy is a high recurrence rate. Symptoms and examination findings reported the quantity and dosage of medications used returned to preoperative levels in 66.6% of patients at month 6, and in all patients except one at the end of the 1st year [120].

Digital periarterial sympathectomy may be considered in patients suffering from critical digital ischemia or persistent ulceration despite aggressive vasodilatory therapy. A long-term retrospective study assessed 35 patients with primary or secondary RP who underwent thoracoscopic sympathectomy: 77% of participants had a positive response. However, symptoms recurred in 60% at a median follow-up of 5 months [121].

Single-port thoracoscopic sympathicotomy (SPTS) is a novel minimally invasive technique compared to conventional sympathectomy [122]. A recent study showed that the single-port procedure is effective in improving hand perfusion in patients with treatment-resistant RP. One month after unilateral single-port thoracoscopic sympathicotomy, the number of RP attacks was reduced and perfusion of the treated hand increased. However, the long-term efficacy and safety profile of this treatment need to be established [123]. 

## 19. Botulinum Toxin Type A

Botulinum toxin type A (BTX-A) is a polypeptide produced by the bacteria *Clostridium botulinum*. It is an acetylcholine release inhibitor in the peripheral nerve endings of the motor plate and sweat glands. It is well established that BTX-A inhibits acetylcholine release, leading to inhibition of neurotransmitter-induced vasoconstriction and relief of other symptoms, such as pain [124,125]. 

Botulinum toxins inhibit macromolecular SNARE complexes, which are involved in vesicle fusion with the plasma membrane, thus preventing neuronal exocytosis [126]. 

A study performed by Medina et al. established botulinum toxin as a safe, accessible, and effective therapeutic alternative for patients with severe RP, allowing those who do not respond to conventional treatments to sustain a good quality of life via annual infiltrations [127]. 

BTX-A is a promising non-surgical treatment modality and/or adjunct for patients who have contraindications to CCBs, PDE-5 inhibitors, and nitrates. It also provides a non-operative therapeutic alternative for patients experiencing chemotherapy-induced RP where mainstay therapies may be contraindicated, thus reducing pain, improving patient quality of life, and slowing disease progression [128]. 

In a study by Nagarajan et al. several patients derived long-term benefits from a single treatment, however in patients with SSc, repeat treatments were required and administered after an average of 6 months [129].

## 20. Riociguat

Riociguat is a first-in-class guanylate cyclase stimulator and may be a promising new treatment for RP. It works by direct stimulation of guanylate cyclase, independent from NO, and additionally via sensitization of guanylate cyclase to endogenous nitric oxide by stabilizing NO–guanylate cyclase binding. As a result, riociguat efficiently stimulates the nitric oxide–soluble guanylate cyclase–cyclic guanosine monophosphate pathway and leads to increased intracellular levels of cyclic guanosine monophosphate. In contrast to PDE-5 inhibitors, the action of riociguat does not depend on endogenous nitric oxide levels [130]. In the pilot study performed by Huntgeburth et al., a single oral dose of riociguat 2 mg was well tolerated in patients with RP and resulted in improved digital blood flow in some patient subsets, with high inter-individual variability [131].

## 21. SSRIs

An improvement in RP symptoms has been reported in patients treated with selective serotonin reuptake inhibitors (SSRIs). A study of 27 patients with SSc revealed that fluoxetine at a dose of 20mg/day was significantly superior to nifedipine in reducing the frequency and severity of RP attacks in patients with SSc [132]. Of note however, exacerbations of RP have also been reported with the use of serotonin reuptake inhibitors treatment [133].

## 22. Treat to Target (T2T) Strategy

At present, the decision to commence treatment and to evaluate response in RP—including the need for dose escalation—is principally based upon clinician–patient discussions regarding symptom severity, perceived effectiveness of the existing/planned interventions, and drug tolerability.

Hughes et al. proposed a five-stage roadmap that may support the development of a treat to target (T2T) strategy for SSc-RP. Significant initial steps are to define the study population and the goals of developing a T2T strategy (stage 1) and to review and shortlist candidate target items (stages 2 and 3, respectively). If agreement regarding feasible targets is not reached at this point, then the goals and purpose need to be refined. Subsequently, a consensus-building exercise among relevant stakeholders would allow the ‘target’ to be defined (stage 4). Ultimately, well-designed studies (stage 5) will be required to investigate the feasibility and treatment benefit of a T2T strategy in patients with SSc-RP. Much can be learned from primary studies of T2T for rheumatoid arthritis, including randomized trials comparing T2T with routine care, and those comparing different treatment approaches (e.g., monotherapy vs. combination therapy) to reach a defined target. Crucial features of these studies were the frequent review of patients, and the clear guidance that existed on how to intensify treatment of patients who had not reached the target [134,135]. 

## 23. Conclusions

An increased understanding of the pathogenesis of Raynaud’s phenomenon is guiding new approaches to treatment. Assessment of peripheral endothelial dysfunction may aid identification of individuals with secondary Raynaud’s phenomenon who are at risk of developing connective tissue diseases, and who may therefore benefit from repeated screening, early initiation of preventative treatments, and risk factor modification. 

Capillaroscopy is of crucial value for the diagnosis and differentiation of primary and secondary Raynaud’s phenomenon.

The presence of digital ulcers implies that intervention with vasodilator therapy is necessary. Early intervention is vital to the treatment of critical ischemia, and calcium channel blockers remain the first line of therapy. Alternatives for severe disease include phosphodiesterase-5 inhibitors and intravenous prostaglandin analogues. The overall benefits of iloprost and alprostadil are comparable without significant differences; however, ease of handling and the lower cost profile favours alprostadil. Sulodexide is a safe rheological drug successfully used as a supportive treatment in RP, resulting in a long-term improvement of capillary flow and a reduction in the frequency of Raynaud’s syndrome relapse. Topical vasodilators, for example 10% nifedipine cream, 10% nitroglycerine gel, and 5% sildenafil cream, may act as adjuvant therapy. Riociguat may be a promising new treatment for Raynaud’s phenomenon; however, this warrants further evaluation. A variety of alternative modalities have also been reported to be effective in the management of RP including botulinum toxin A, and sympathectomy, or single-port thoracoscopic sympathectomy. 

Treat to target strategies may optimize treatment approaches for Raynaud’s phenomenon and herald the emergence of disease-modifying vasodilator therapies for systemic sclerosis-related digital vasculopathy. 

## Figures and Tables

**Table 2:** Raynaud’s phenomenon—proposed laboratory tests [16,17]. TPO—thyroid peroxidase; TG—thyroglobulin; ANA—antinuclear antibody.

Raynaud’s Phenomenon—Proposed Laboratory Tests
Complete blood count Erythrocyte sedimentation rate Thyroid function tests: ▪ Thyroid-stimulating hormone (TSH) ▪ Thyroxine (T4) ▪ TPO antibodies ▪ TG antibodies Anti-HCV Rheumatoid factor C-reactive protein Cryoglobulins Creatinine Urea ANA test Protein electrophoresis Urinalysis

**Table 3:** Pharmacotherapy options in management of Raynaud’s phenomenon. A-level recommendation is based on consistent and good-quality patient-oriented evidence; B-level recommendation is based on inconsistent or limited-quality patient-oriented evidence; C-level recommendation is based on consensus, usual practice, opinion, disease-oriented evidence, or case series for studies of diagnosis, treatment, prevention, or screening.

Group of Drugs	Medication	Dose	Strength of Recommendation
Calcium Channel Antagonists	nifedipine	10–20 mg 3× daily or extended-release tablets	A
	nifedypine SR	30–120 mg daily	
Phosphodiesterase Type 5 Inhibitors	sildenafil	50–100 mg 2× daily (the suggested starting dose is 12.5 mg/day, to be increased gradually depending on tolerability)	A
Prostaglandin Analogs	alprostadil (*i.v*. infusions)	pulses of 20–60 mg every 4–6 weeks	A
	iloprost (*i.v.* infusions/*p.o*.)	0.5–3 ng/kg/min (*i.v.*) for 3–5 consecutive days every 6–8 weeks or 50–150 μg 2× daily (*p.o.*)	A
	epoprostenol (*i.v*. infusions)	2 ng/kg/min in intermittent infusions of 5 to 6 hours’ duration	A
First-in-class Guanylate Cyclase Stimulator	riociguat	2 mg single oral dose	C
Selective Serotonin Reuptake Inhibitors (SSRIs)	fluoxetine	20 mg/day	C
Endothelin Receptor Antagonists	bosentan	125 mg twice a day following initial dosage of 62.5 mg twice a day	C
Angiotensin II receptor blockers	losartan	25 mg once daily to 100 mg once daily	C
Mixture of glycosaminoglycans composed of dermatan sulfate and fast moving heparin	sulodexide	3–4 day cycle of intravenous sulodexide, at 600 LSU twice a day every 4–6 weeks	C
3-hydroxy-3-methylglutaryl coenzyme A (HMG-CoA) reductase inhibitors	atorvastatin	40 mg/day	C
Botulinum toxin	type A	Dose dependent vasodilation with 10–100 units injections	C
Topical vasodilators	Nifedipine Nitroglycerin sildenafil	10% nifedipine cream 10% nitroglycerin gel 5% sildenafil cream	C
Surgical treatment	sympathectomy/arterial reconstruction		C

## Data Availability

The study did not report any data.
